# Antibacterial household products: cause for concern.

**DOI:** 10.3201/eid0707.017705

**Published:** 2001

**Authors:** S B Levy

**Affiliations:** Tufts University School of Medicine, Boston, Massachusetts 02111, USA. stuart.levy@tufts.edu

## Abstract

The recent entry of products containing antibacterial agents into healthy households has escalated from a few dozen products in the mid-1990s to more than 700 today. Antibacterial products were developed and have been successfully used to prevent transmission of disease-causing microorganisms among patients, particularly in hospitals. They are now being added to products used in healthy households, even though an added health benefit has not been demonstrated. Scientists are concerned that the antibacterial agents will select bacteria resistant to them and cross-resistant to antibiotics. Moreover, if they alter a person's microflora, they may negatively affect the normal maturation of the T helper cell response of the immune system to commensal flora antigens; this change could lead to a greater chance of allergies in children. As with antibiotics, prudent use of these products is urged. Their designated purpose is to protect vulnerable patients.


### Antibacterial Household Products: Cause for Concern

**Stuart B. Levy**
Tufts University School of Medicine, Boston, Massachusetts, USA

The recent entry of products containing antibacterial agents into healthy households has escalated from a few dozen products in the mid-1990s to more than 700 today. Antibacterial products were developed and have been successfully used to prevent transmission of disease-causing microorganisms among patients, particularly in hospitals. They are now being added to products used in healthy households, even though an added health benefit has not been demonstrated. Scientists are concerned that the antibacterial agents will select bacteria resistant to them and cross-resistant to antibiotics. Moreover, if they alter a person's microflora, they may negatively affect the normal maturation of the T helper cell response of the immune system to commensal flora antigens; this change could lead to a greater chance of allergies in children. As with antibiotics, prudent use of these products is urged. Their designated purpose is to protect vulnerable patients.

Antibiotics are critical to the treatment of bacterial infections. However, after years of overuse and misuse of these drugs, bacteria have developed antibiotic resistance, which has become a global health crisis (1, 2). The relatively recent increase of surface antibacterial agents or biocides into healthy households may contribute to the resistance problem. The antibacterial substances added to diverse household cleaning products are similar to antibiotics in many ways. When used correctly, they inhibit bacterial growth. However, their purpose is not to cure disease but to prevent transmission of disease-causing microorganisms to noninfected persons. Like antibiotics, these products can select resistant strains and, therefore, overuse in the home can be expected to propagate resistant microbial variants (3-6). Moreover, these agents, like antibiotics, are not cure-alls but have a designated purpose. Whereas antibiotics are designed to treat bacterial (not viral) infections, antibacterial products protect vulnerable patients from infectious disease-causing organisms. Neither are demonstrably useful in the healthy household.

### Proliferation of Antibacterial Products

Seven years ago, only a few dozen products containing antibacterial agents were being marketed for the home. Now more than 700 are available. The public is being bombarded with ads for cleansers, soaps, toothbrushes, dishwashing detergents, and hand lotions, all containing antibacterial agents. Likewise, we hear about "superbugs" and deadly viruses. Germs have become the buzzword for a danger people want to eliminate from their surroundings. In response to these messages, people are buying antibacterial products because they think these products offer health protection for them and their families. Among the newer products in the antibacterial craze are antibacterial window cleaner and antibacterial chopsticks. Antibacterial agents are now in plastic food storage containers in England. In Italy, antibacterial products are touted in public laundries. In the Boston area, you can purchase a mattress completely impregnated with an antibacterial agent. Whole bathrooms and bedrooms can be outfitted with products containing triclosan (a common antibacterial agent), including pillows, sheets, towels, and slippers.

### Development of Resistance

Bacteria are not about to succumb to this deluge, however. Through mutation, some of their progeny emerge with resistance to the antibacterial agent aimed at it, and possibly to other antimicrobial agents as well (4). Laboratory-derived mutants of *Pseudomonas stutzeri* with resistance to the cationic biocide chlorhexidine were also cross-resistant to antibiotics (nalidixic acid, erythromycin, and ampicillin) (7). In a recent study, 7% of *Listeria monocytogenes* strains isolated from the environment and food products showed resistance to quaternary ammonium compounds (8).

Laura McMurry in my laboratory group conducted experiments to determine whether triclosan had a particular cellular site for its antibacterial activity. She used a classic genetic technique, the isolation of resistant mutants of *Escherichia coli*, to identify its possible target. Surprisingly, finding the cellular site proved easy. In fact, mutants appeared with low, medium, and high-level resistance (3). They all had a mutation in one gene, the *fabI* gene (3) (Table 1). This finding indicated that triclosan had a target for the enoyl reductase essential in fatty acid biosynthesis. In the presence of triclosan, or a known FabI inhibitor (diazoborine), fatty acid biosynthesis was inhibited, whereas the antibiotics chloramphenicol or ciprofloxacin with other targets had little effect on fatty acid

Table 1. Selection of *Escherichia coli* with triclosan resistance (3)

| *E. coli* | MIC (µg/ml) | Change (fold) | Mutated gene |
|---|---|---|---|
| AG100 | 0.05 | 1.0 | —— |
| AG100-1 | 0.20 | 4.0 | *fabI* (F203L) |
| AG100-2 | 1.90 | 40.0 | *fabI* (M159T) |
| AG100-3 | 25.00 | 500.0 | *fabI* (G93V) |

Address for correspondence: Stuart B. Levy, Center for Adaptation Genetics and Drug Resistance, Tufts University School of Medicine, 136 Hansen Ave., Boston, MA 02111 USA; fax: 617-636-0458; e-mail: stuart.levy@tufts.edu biosynthesis (Table 2). In comparison with the wild-type *E. coli*, the mutant required up to 100 times more triclosan to show even minimal inhibition of fatty acid biosynthesis (3).

Table 2. Effect of various drugs on fatty acid/lipid synthesis in intact cells

| Strain | Drug | µg/ml | % inhibition |
|---|---|---|---|
| AG100 | Triclosan | 0.24 | 92 |
| (wild-type) | Diazaborine | 8.0 | 93 |
| | Chloramphenicol | 13.0 | 19 |
| | Ciprofloxacin | 0.045 | 2 |
| AGT11 | Triclosan | 0.24 | 2 |
| (G93V) | Triclosan | 1.4 | 7 |
| | Triclosan | 8.6 | 37 |
| | Triclosan | 25.9 | 75 |

One might argue that the high concentration of triclosan usually found in soap, e.g. 2,500 µg/ml, is enough to kill even resistant strains. We examined this question by testing triclosan activity in a commercial soap. To achieve a 90% death rate, wild-type *E. coli* required exposure to 150 µg/mL of triclosan in soap for 2 hours at 37°C. Two to four times that amount was required by the mutant. By itself, triclosan was more active, killing *E. coli* at 6 µg/ml, and there was an even greater difference between the amounts required to kill wild-type and mutant *E. coli*. The soap seemed to decrease triclosan's effectiveness (Table 3). The mutant *E. coli* strains are truly resistant and would survive in triclosan-treated soaps diluted with as little as 3 parts water. Most importantly, the time, temperature, and amount needed to kill the bacteria greatly exceeded the average 5-second hand washing performed by most people.

Table 3. Effect of triclosan in soap on *Escherichia coli*[a]

| Strain | In nutrient broth | In nutrient broth with commercial soap |
|---|---|---|
| AG100[b] | 6 | 150 |
| AGT11[c] | >32 | 300-600 |

[a] Amount (µg/ml) needed to kill 90% of *E. coli* (2 h at 37°C).
[b] Wild-type
[c] Mutant

The finding of a mutation in the *fabI* gene led to a study of its homologue, *inhA*, the gene for one of the proposed targets of isoniazid, an anti-tuberculosis drug. Whether selected in triclosan or isoniazid, mutants of *Mycobacterium smegmatis* showed cross-resistance to both drugs via a mutation in the *inhA* gene (Table 4) (9). Moreover, triclosan-resistant *E. coli* mutants also showed resistance to an experimental antibiotic, diazoborine (3). Other drugs currently under development may target *fabI*; these potentially new antibiotics may also be affected by triclosan resistance.

The data clearly suggest that antibacterial agents will have an impact on the environmental flora and on resistance emergence. For instance, use of triclosan could select bacteria which have intrinsic resistance to the chemical. Some gram-positive bacteria such as *Enterococcus faecalis* and *Streptococcus pneumoniae*, which do not have *fabI*, have a related enoyl reductase gene, *fabK* (10). The *fabK* gene in those organisms is naturally resistant to triclosan, so

Table 4. Mutations in InhA confer triclosan resistance in *Mycobacterium smegmatis* (9)

| Strain | Selected on[a] | InhA mutation | Relative MIC (in 7H9 medium) TRC | INH |
|---|---|---|---|---|
| $mc^2155$ (wt) | — | None | 1.0 | 1.0 |
| MT 1 | TRC | M161V | 6.3 | 8.5 |
| MT 9 | TRC | M103T | 6.3 | 1.2 |
| MT 17 | TRC | A124V | 5.8 | 2.0 |
| $mc^2651$ | INH | S94A | 6.3 | 22.0 |
| $mc^2155$ (p::inhA) | — | None | 6.3 | >64 |

[a]TRC, triclosan; INH, isoniazid triclosan usage can potentially enhance their growth at the expense of susceptible strains. At the American Society for Microbiology meetings in May 2000, a number of papers described the isolation of bacteria resistant to triclosan or to other antibacterial agents (11-13).

The other known mechanism by which bacteria resist these drugs is by pumping them out of the cell by an efflux mechanism. The key genes in *E. coli* involved in this form of resistance are a regulatory gene, *marA*,and an efflux gene complex, *acrAB* (14). *MarA* is a component of a multiple antibiotic resistance locus, *marRAB*. When *marA* is activated, the cell becomes resistant to antibiotics, oxidative stress agents, organic solvents, and antibacterial agents (14). Over 60 different genes are affected when *marA* is overexpressed in *E. coli*, indicating a very large regulon (15). Strains that overproduce the marA or soxS protein (which is a *marA* homologue) upregulate the AcrAB multidrug efflux pump which pumps out pine oils, organic solvents, triclosan, quaternary ammonium compounds, chloroxanol, and chlorhexidine (4). Triclosan is also a substrate for multidrug efflux pumps in *Pseudomonas aeruginosa* (16).

Efflux pumps can affect antibiotic efficacy in a number of ways. A triclosan-resistant mutant of *E. coli* does not lyse easily in the presence of triclosan, making the strain difficult to kill. Triclosan lyses the wild-type cell (AG100) at about 8 µg/ml, but the mutant AGT11 requires at least four times that amount (=32 µg/ml) (Table 5). When the *acrAB* gene locus is deleted from the wild-type cell, lysis occurs at a lower concentration, i.e., 3 to 4 µg/ml. More importantly, with removal of the AcrAB pump, the mutant bacteria and the wild-type cells were killed by the same amount of triclosan, i.e., 3 to 4 µg/ml, despite residual *fabI* resistance in the mutant to the growth inhibitory action of triclosan (Table 5). Therefore, the normal expression of a multidrug efflux pump in *E. coli* is critical to the activity of triclosan.

Table 5. Effect of triclosan in liquid culture on growth and lysis of *Escherichia coli* strains with and without AcrAB efflux pump (4)

| Strain | Characteristics | Concentration (µg/ml) that: Inhibited growth 50% | (90%) | Caused lysis |
|---|---|---|---|---|
| AG100 | Wild-type | 0.15 | (0.60) | 8 |
| AG100A | AG100 *acrAB::kan* | 0.02 | (0.05) | 3-4 |
| AGT11 | AG100 *fabI* (G93V) | 13.0 | (>32) | >32 |
| AGT11K | AGT11 *acrAB::kan* | 1.3 | (2.10) | 3-4 |

Mar mutants generally express low levels of antibiotic resistance and are precursors to mutants with high-level antibiotic resistance (14). We have identified clinical strains of *E. coli* that are resistant to triclosan because they are also Mar mutants (4). From these and other data, selection for Mar mutants can potentially occur by antibiotics or by antibacterial agents.

### Consequences of Resistance

Community-acquired methicillin-resistant *Staphylococcus aureus* (cMRSA) has become an increasing problem worldwide. These community-derived strains show an antibiotic susceptibility profile that is markedly different from hospital-acquired MRSA. cMRSA strains are chiefly resistant to the beta-lactam antibiotics (penicillins and cepholosporins). Interesting laboratory findings suggest a link between this resistance in cMRSA and the use of antibacterial products. Investigators in Japan selected MRSA mutants with a twofold higher minimal inhibitory concentration for benzalkonium chloride (5 to 10 µg/ml) (17). Resistance to methicillin and to a number of cephalosporins and penicillins dramatically increased with this mutation (Table 6), but susceptibility to other antibiotics was essentially unchanged. The laboratory mutants, in fact, mirror the phenotype of the MRSA that has emerged in the community. Is there a connection? The findings warrant further study.

Table 6. Antibiotic susceptibility of methicillin-resistant *Staphylococcus aureus* and benzalkonium chloride-resistant derivatives[a]

| | MRSA[b] | | |
|---|---|---|---|
| Antibiotic | Parent | BZ-R-1 | BZ-R-2 |
| Oxacillin | 16.0 | 512 | 512 |
| Cloxacillin | 0.5 | 256 | 512 |
| Moxalactam | 64.0 | 256 | 1024 |
| Flomoxef | 8.0 | 128 | 128 |
| Cefmetazole | 8.0 | 128 | 64 |
| Cephalothin | 64.0 | 128 | 128 |
| Ampicillin | 16.0 | 32 | 32 |
| Chloramphenicol | 4.0 | 4 | 4 |
| Ofloxacin | 8.0 | 32 | 32 |
| Tetracycline | 128.0 | 128 | 128 |
| Benzalkonium chloride | 5.0 | 10 | 10 |

[a]Adapted from (17).
[b]MIC (µg/ml) (BZ-R = parent resistant to benzalkonium chloride)

Antibacterial agents and antibiotics share the same resistance problem. Resistance will certainly increase as the drug persists, especially at low levels (e.g., residues) for long periods of time. Of course, that concern is irrelevant with substances that do not leave residues (e.g., alcohols, bleaches, and peroxides). No current data demonstrate any health benefits from having antibacterial-containing cleansers in a healthy household. However, use of these products may change the environmental microbial flora.

Unfortunately, the antibacterial indulgence is coincident with the trend toward shorter hospital stays. An estimated 5% of hospital patients in Massachusetts go home for continued care, often with intravenous parenteral drugs. For these vulnerable patients, the use of antibacterial products protects them from disease caused by commensal as well as pathogenic bacteria. A cause for concern now is that homes, which are becoming end-of-therapy quarters for patients, may be becoming havens for "hospital-like" bacteria as well.

### The Antibacterial Products-Allergy Link

Besides resistance, the antibacterial craze has another potential consequence. Reports are mounting about a possible association between infections in early childhood and decreased incidence of allergies (18). In expanding this "hygiene hypothesis," some researchers have found a correlation between *too much* hygiene and *increased* allergy (18-21). This hypothesis stems from studies that revealed an increased frequency of allergies, cases of asthma, and eczema in persons who have been raised in an environment overly protective against microorganisms. In one rural community, children who grew up on farms had fewer allergies than did their counterparts who did not live on farms (19). Graham Rook, University College, London, has likened the immune system to the brain. You have to exercise it, that is, expose it to the right antigenic information so that it matures correctly. Excessive hygiene, therefore, may interfere with the normal maturation of the immune system by eliminating the stimulation by commensal microflora (20).

For normal maturation, the immune system must be stimulated to achieve the right balance between the T-helper 1 (TH-1) cells providing cellular immunity and the TH-2 cells promoting antibody production. When investigators examined people with allergies and eczema, they noted an imbalance between TH-2 and TH-1 activities as compared with the mechanisms in control groups. In those with allergies, antibody production predominated over cell-mediated responses. Other studies showed a correlation between the presence of an immune response to organisms contracted by the oral-fecal route and decreased likelihood of atopy (21). In those persons who demonstrated a prior exposure to one, two, or all three of the organisms tested (*Toxoplasma gondii*, *Helicobacter pylori*, hepatitis A virus), the odds ratio for allergy became substantially lower than that seen in the control group (21). This correlation was not found for prior contact with organisms causing infections by other routes (e.g., mumps, measles, varicella). The authors concluded that "hygiene and a westernized, semi-sterile diet may facilitate atopy by influencing the overall pattern of commensals and pathogens that stimulate the gut-associated lymphoid tissue …" (21). Of note, children vaccinated with bacillus Calmette-Guérin appeared to be protected as well against atopy (22), and this finding was also related to stimulation of the TH-1 response. The combined data led one group to conclude that an "antigenically rich (dirty) environment may be essential for normal immune maturation preventing atopic disease" (23).

Antibiotics may also be implicated in the hygiene hypothesis. Because they eliminate common bacteria, antibiotics may cause the same consequence as too much hygiene. Some infants begin to get antibiotics as soon as a few days after birth. They mature in an antibiotic-laden environment. What antigens do they confront daily? What kind of immune response are they developing?

We must think not just in terms of resistance but also in terms of the changes in the microbial ecology of our infants and our homes. We exist in the bacterial world, not bacteria in ours. Unfortunately, we believe that we can rid ourselves of bacteria when, in fact, we cannot. Instead, we should "make peace" with them. Although we need to control pathogens when they cause disease, we do not have to engage in a full-fledged "war" against the microbial world. Improved antibiotic use, including shorter treatments and removal of improper usage, will encourage the return of antibiotic-susceptible, commensal flora and return the environment to what it was before the antibiotic/antibacterial onslaught.

A new approach focusing on commensals has been initiated through an agreement between the Alliance for Prudent Use of Antibiotics (APUA) (www.apua.org) and the University of Illinois, funded by the National Institute of Allergy and Infectious Disease. This initiative, entitled ROAR (Reservoirs of Antibiotic Resistance; http://www.roar.apua.org) focuses on monitoring and managing the commensal bacteria that harbor pools of resistance genes that can be passed on to pathogens. Through education, APUA strives to foster control of pathogens without decimation of the non-pathogens. In this goal, prudent use applies to both antibiotics and antibacterial products.

Research in this laboratory has been supported through grants from the National Institutes of Health.

Dr. Levy is director of the Center for Adaptation Genetics and Drug Resistance, professor of molecular biology and microbiology, and of medicine, Tufts University School of Medicine, Boston. He is past president of the American Society for Microbiology and President of the Alliance for Prudent Use of Antibiotics.

### References

1. Neu HC. The crisis in antibiotic resistance. Science 1992;257:1064-73.
2. Levy SB. The antibiotic paradox. How miracle drugs are destroying the miracle. New York: Plenum; 1992.
3. McMurry LM, Oethinger M, Levy SB. Triclosan targets lipid synthesis. Nature 1998;394:531-2.
4. McMurry LM, Oethinger M, Levy SB. Overexpression of *marA*, *soxS* or *acrAB* produces resistance to triclosan in *Escherichia coli*. FEMS Microbiol Lett 1998;166:305-9.
5. Suller MT, Russell AD. Triclosan and antibiotic resistance in *Staphylococcus aureus*. J Antimicrob Chemother 2000;46:11-8.
6. Hoang TT, Schweizer HP. Characterization of *Pseudomonas aeruginosa* enoyl-acyl carrier protein reductase (FabI): a target for the antimicrobial triclosan and its role in homoserine lactone synthesis. J Bacteriol 1999;181:5489-97.
7. Russell AD, Tattawasart U, Maillard J-Y, Furr JR. Possible link between bacterial resistance and use of antibiotics and biocides. Antimicrob Agents Chemother 1998;42:2151.
8. Mereghetti L, Quentin R, Marquet-van der Mee N, Audurier A. Low sensitivity of *Listeria monocytogenes* to quaternary ammonium compounds. Appl Environ Microbiol 2000;66:5083-6.
9. McMurry LM, McDermott PF, Levy SB. Genetic evidence that InhA of *Mycobacterium smegmatis* is a target for triclosan. Antimicrob Agents Chemother 1999;43:711-3.
10. Heath RJ, Roch CO. A triclosan-resistant bacterial enzyme. Nature 2000;406:145-6.
11. Radosti-Slater C, Aller GV, DeWolf W, Greenwood R, Nicholas R, Payne D, et al. Mode of action of triclosan in *S. aureus* [abstract]. American Society for Microbiology annual meeting, Los Angeles, California, 2000 May 21-24. Abstract 101, p. 26.
12. Meade MJ, Callahan TM. Unique mechanism of triclosan resistance identified in environmental isolates [abstract]. American Society for Microbiology annual meeting, Los Angeles, California, 2000 May 21-24. Abstract 73, p. 19.
13. Suzangar S, Allison DG, Gilbert P. An evaluation of biocide-containing materials for their surface colonization-resistance and other properties [abstract] American Society for Microbiology annual meeting, Los Angeles, California, 2000 May 21-24. Abstract 53, p. 17.
14. Alekshun MN, Levy SB. The *mar* regulon: multiple resistance to antibiotics and other toxic insults. Trends Microbiol 1999;7:410-3.
15. Barbosa T, Levy SB. Differential expression of over 60 chromosomal genes in *Escherichia coli* by constitutive expression of MarA. J Bacteriol 2000;182:3467-74.
16. Chuanchuen R, Beinlich K, Schweizer HP. Multidrug efflux pumps and triclosan resistance in *Pseudomonas aeruginosa* [abstract]. American Society for Microbiology annual meeting, Los Angeles, California, 2000 May 21-24. Abstract 31, p. 8.
17. Akimitsu N, Hamamoto H, Inoue R, Shoji M, Akamine A, Takemori K, et al. Increase in resistance of methicillin-resistant *Staphylococcus aureus* to beta-lactams caused by mutations conferring resistance to benzalkonium chloride, a disinfectant widely used in hospitals. Antimicrob Agents Chemother 1999;43:3042-3.
18. Strachan DP. Hay fever, hygiene, and household size. BMJ 1989;299:1259-60.
19. Braun-Fahrländer CH, Gassner M, Grize L, Neu U, Sennhauser FH, Varonier HS, et al. Prevalence of hay fever and allergic sensitization in farmer's children and their peers living in the same rural community. Clin Exp Allergy 1999;29:28-34.
20. Rook GAW, Stanford JL. Give us this day our daily germs. Immunol Today 1998;19:113-6.
21. Matricardi PM, Rosmini F, Riondino S, Fortini M, Ferrigno L, Rapicetta M, et al. Exposure to foodborne and orofecal microbes versus airbone viruses in relation to atopy and allergic asthma: epidemiological study. BMJ 2000;320:412-7.
22. Aaby P, Shaheen SO, Heyes CB, Goudiaby A, Hall AJ, Shiell AW, et al. Early BCG vaccination and reduction in atopy in Guinea-Bissau. Clin Exp Allergy 2000;30:644-50.
23. Folkerts G, Walzl G, Openshaw PJM. Do common childhood infections "teach" the immune system not to be allergic? Immunol Today 2000;21:118-20.
